# *In-situ* terahertz optical Hall effect measurements of ambient effects on free charge carrier properties of epitaxial graphene

**DOI:** 10.1038/s41598-017-05333-w

**Published:** 2017-07-11

**Authors:** Sean Knight, Tino Hofmann, Chamseddine Bouhafs, Nerijus Armakavicius, Philipp Kühne, Vallery Stanishev, Ivan G. Ivanov, Rositsa Yakimova, Shawn Wimer, Mathias Schubert, Vanya Darakchieva

**Affiliations:** 1 Department of Electrical and Computer Engineering, University of Nebraska-Lincoln, Lincoln, Nebraska 68588-0511 USA; 2 Terahertz Materials Analysis Center, Department of Physics, Chemistry and Biology, Linköping University, SE 581 83 Linköping, Sweden; 3 Department of Physics and Optical Science, University of North Carolina, Charlotte, North Carolina 28223 USA; 4 Semiconductor Materials Division, Department of Physics, Chemistry and Biology, Linköping University, SE 581 83 Linköping, Sweden; 5 Leibniz-Institut für Polymerforschung Dresden e.V., Dresden, 01069 Germany

## Abstract

Unraveling the doping-related charge carrier scattering mechanisms in two-dimensional materials such as graphene is vital for limiting parasitic electrical conductivity losses in future electronic applications. While electric field doping is well understood, assessment of mobility and density as a function of chemical doping remained a challenge thus far. In this work, we investigate the effects of cyclically exposing epitaxial graphene to controlled inert gases and ambient humidity conditions, while measuring the Lorentz force-induced birefringence in graphene at Terahertz frequencies in magnetic fields. This technique, previously identified as the optical analogue of the electrical Hall effect, permits here measurement of charge carrier type, density, and mobility in epitaxial graphene on silicon-face silicon carbide. We observe a distinct, nearly linear relationship between mobility and electron charge density, similar to field-effect induced changes measured in electrical Hall bar devices previously. The observed doping process is completely reversible and independent of the type of inert gas exposure.

## Introduction

Two dimensional materials are a new class of materials that attracted significant interest due to their unique electronic, optical, and mechanical properties. Many properties differ dramatically from the bulk substances. Examples include silicene, molybdenum disulfide, hexagonal boron nitride, and graphene^1–4^. These materials have wide spread applications such as high-frequency electronics, sensing elements, mechanically durable and lightweight materials, medicine, energy storage, and more. Common to all two dimensional materials is their extremely large surface to volume ratio, which make them very susceptible to substrate and ambient gas adsorption effects. Graphene, one of the most studied two dimensional materials, has been shown to substantially change in its properties as a function of substrate, substrate polarity, and ambient media (gases, liquids)^5–11^. Ambient induced doping is observed for many types of graphene, for instance epitaxial graphene on SiC^5–7^, exfoliated graphene on SiO_2_^8^, and chemical vapor deposition (CVD) grown graphene^9^. However, the effect of ambient doping on the free charge carrier mobility is rarely explored, mostly due to the lack of experimental techniques capable of independently assessing carrier density and mobility without further modifying graphene. For instance standard electrical Hall effect measurements require contacts and Hall bar fabrication, which involve multiple processing steps that may modify graphene properties. In this work, ambient effects on the free charge carrier density and mobility of epitaxial graphene grown by Si-sublimation on the Si-face (0001) of 4H-SiC is studied as an example^12^.

This type of graphene typically exhibits n-type conductivity due to a complex interaction with the Si-face SiC substrates via the buffer layer^13, 14^. The intrinsic electron doping of pristine monolayer epitaxial graphene on Si-face SiC was found to be on the order of 10^13^ cm^−2^ ^6^. The polarity of the Si-face SiC also gives rise to high hydrophilicity, which is relatively unchanged with addition of a graphene monolayer^15^. Similar to other two dimensional materials, graphene’s properties are also extremely sensitive to adsorbed gas molecules^5–7, 16, 17^. Previous studies of the electrical properties of epitaxial graphene on SiC as a function of the ambient conditions employ contact-based techniques or Kelvin probe approaches and typically do not report changes in *N*_s_ and *μ* simultaneously. For example, the authors in Ref. 5 use a 4-point probe device to perform resistance and thermo-electric power measurements during various phases of gas exposure and annealing cycles. Reference 6 reports on characterization using a Hall bar device and Scanning Kelvin Probe Microscopy (SKPM), and which do not provide changes in *N*_s_ and *μ* simultaneously. In order to fully understand the ambient doping and scattering mechanisms in epitaxial graphene, accurate information on the free charge carrier parameters as a function of the ambient conditions is required. Here we report on the first *in-situ*, contactless determination of the majority free charge carrier type, *N*_s_, and *μ* using terahertz-frequency optical Hall effect measurements (THz-OHE)^18–20^. We expose a monolayer graphene sample to various gases and report the results of the best-match model data analysis from optical Hall effect measurements. Along with *N*_s_ and *μ*, the THz-OHE signal allows us to determine the majority free charge carrier type (electrons). This is important information since the majority carrier type of epitaxial graphene on Si-face SiC can vary from sample to sample^16, 21, 22^. We find that exposure to ambient causes significant changes in the free charge carrier properties sheet carrier concentration *N*_s_ and mobility *μ* for epitaxial graphene on SiC.

## Results and Discussion

**Figure 1:**
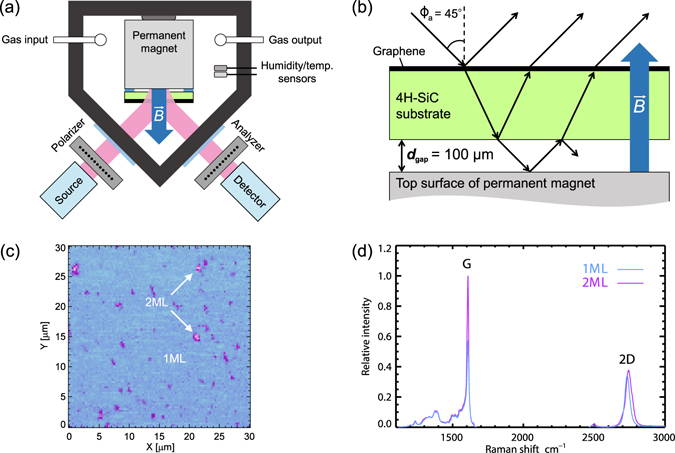
(**a**) Schematic of single-layer epitaxial graphene on SiC substrate located on top of a permanent magnet within a sealed gas chamber with optical ports for polarized THz radiation, and THz ellipsometer setup. (**b**) Schematic of the cavity-enhancement of the THz optical Hall effect using a resonant cavity between the sample and magnet surface. (**c**) Representative micro-reflectance map of the graphene surface. (**d**) Representative micro-Raman spectra of 1 ML and 2 ML sample areas.

Figure 1a details a conceptual drawing of the sample arrangement within the gas flow cell. Ellipsometric data are measured at oblique incidence as a function of time for various gas exposure phases. Figure 1b depicts the scheme of the optical Hall effect arrangement, where an external cavity is added. The cavity significantly enhances the optical Hall effect in two-dimensional charge carrier densities^18^. The sample investigated here consists of 99% monolayer (ML) graphene and 1% bilayer (BL) inclusions as illustrated in a representative microscopic reflectance-converted-thickness map in Fig. 1c. Reflectivity and low-energy electron microscopy mapping, and scan lines confirm the thickness homogeniety of the epitaxial graphene sample over the entire surface area of 10 mm × 10 mm. A representative micro-Raman spectra for 1 ML and 2 ML sample areas is shown in Fig. 1d. See the Methods section for further details on the experimental setup.

### *In-situ* optical Hall effect gas exposure monitoring

**Figure 2:**
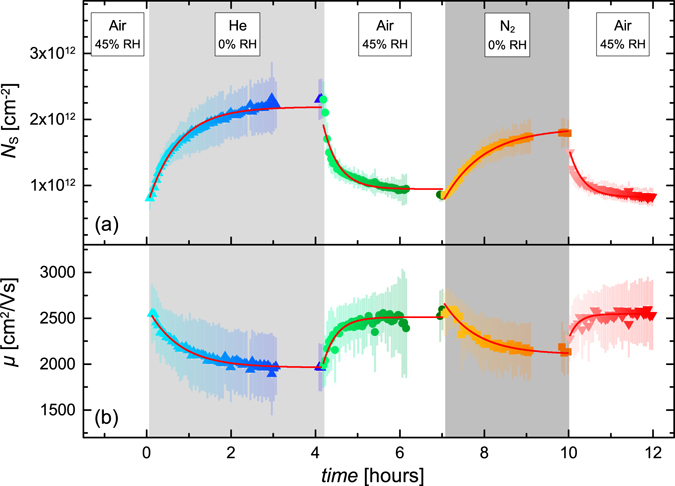
Best-match model results for sheet carrier density *N*_s_ and mobility *μ* as a function of time. The shaded regions correspond to exposure to different types of gas at various relative humidities (RH). The flow rate of 0.5 liters/minute at normal pressure is constant for all exposure phases. Error bars for the best-match model parameters are shown here for each data point as vertical lines. Solid lines depict single-process exponential decay functions, for which rate constants and equilibrium parameters are given in the text.

Figure 2 shows the *in-situ* optical Hall effect best-match model results for sheet carrier density *N*_s_ and mobility *μ* as a function of gas exposure for the ML graphene sample. After growth, the sample was exposed to normal ambient conditions (air measured with relative humidity (RH) of 45%) for approximately 15 minutes, and then mounted in the gas flow cell. During the experiment, large dynamic changes in *N*_s_ and *μ* are observed when changing the gas type. It can be seen that nitrogen and helium exposure (both 0% RH) increases *N*_s_ while ambient exposure (45% RH) decreases *N*_s_. This behavior is consistent with previous results on epitaxial graphene on SiC^6, 16^. The change in *N*_s_ from air exposure can be explained by a redox reaction at the surface of the graphene involving various environmental gases, which results in electron withdrawal^5^. Exposure to an inert gas, such as nitrogen or helium, is thought to cause the doping agents at the graphene surface to desorb, which reverses the electron withdrawal.

The prolonged exposure of single-layer graphene to inert gases drastically changes *N*_s_ and *μ*. We observe an approximate single process exponential decay with mean life time *τ* for both quantities, where the exposure to helium results in faster changes (*τ*_He _≈  45 min) than the exposure to nitrogen (${\tau }_{{{\rm{N}}}_{2}}$ ≈ 50 min). The exposure to air results in electron reduction at a rate (*τ*_Air_ ≈ 20 min) faster than the observed changes during the exposure to the inert gases. We estimate the equilibrium electron density (*N*_s,∞_) and mobility (*μ*_s,∞_) parameters for infinite exposure time of helium, nitrogen, and air as 2.2 × 10^12^ cm^−2^ and 1960 cm^2^/Vs, 1.9 × 10^12^ cm^−2^ and 2100 cm^2^/Vs, and 8.8 × 10^11^ cm^−2^ and 2530 cm^2^/Vs, respectively. There is a noticeable difference in *N*_s,∞_ and *μ*_s,∞_ for He and N_2_ exposure. The cause of this difference is unknown at this point. The difference may be explained by more effective impurity desorption from He compared to N_2_, or in different modifications of the graphene-SiC interaction.

One possible scenario explaining the observed time evolution of *N*_s_ shown in Fig. 2 is an ambient acceptor doping redox reaction at the graphene surface involving O_2_, H_2_O, and CO_2_^5^. To summarize this previously proposed mechanism, first thin films of water form at surfaces when exposed to ambient. These films contain dissolved CO_2_, which reacts with water causing an excess of H^+^. O_2_ dissolved in the water film reacts with H^+^ and electrons borrowed from the graphene and forms additional water molecules, thereby chemically acceptor doping the graphene. While this reaction may not be the only possible chemical process, it is described as the most dominant^23^. In principle, it is also possible that the observed changes in *N*_s_ and *μ* may be related to a modification of the Van der Waals interaction between Si-C and C-C at the SiC-graphene interface. However, exposure to ambient and different inert gases of bare SiC substrate and a sample containing only a buffer layer grown at the same conditions as the epitaxial graphene show no change in the THz-OHE signals. We also note that both SiC substrate and the buffer sample do not produce any free charge carrier related response.

### Mobility and conductivity dependence on carrier density

**Figure 3:**
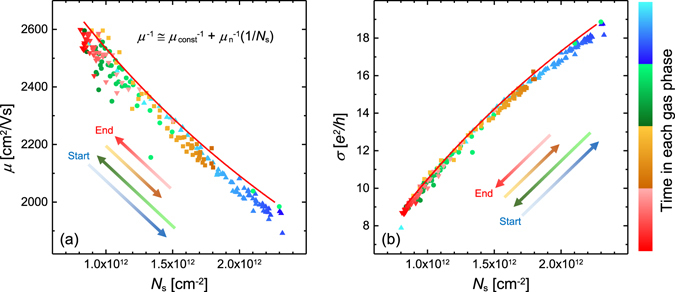
Panel (a) shows mobility *μ* versus sheet density *N*_s_ for all data in Fig. 2. Panel (b) shows conductivity σ versus *N*_s_, where σ is expressed in quantum units (*e* ^2^/*h*). Red lines show the best-match model fit for *μ*(*N*_s_) using the equation *μ* ^−1^ ≈ *μ*_const_^−1^ + *N*_s_/*a*, where *a* is the constant fit parameter﻿. Arrows indicate directions of time evolution. Colors and symbols identify phases of gas exposure as in Fig. 2.

In order to investigate the free charge carrier scattering mechanisms, which are affected by the observed chemical acceptor doping of graphene, *μ* and conductivity σ are plotted as a function of *N*_s_ in Fig. 3. Figure 3a shows the results in Fig. 2 as *μ* versus *N*_s_. Figure 3b depicts the results from Fig. 2 now as σ versus *N*_s_, where σ = *N*_s_ *eμ* and *e* is the electron charge^24^. Interestingly, the same nearly linear dependence of *μ* versus *N*_s_ with time is observed regardless of the type of gas exposure. Since different scattering mechanisms produce different functional dependencies of mobility versus density, one might anticipate that exposure to different gases would result in different traces of *μ* versus *N*_s_. However, the observed common traces for He and N_2_ suggest that the scattering mechanisms which cause variations in *μ* are similar. The common traces for inert and ambient gases suggest that inert gases reverse the scattering mechanism influence from air exposure.

In an attempt to apply a model for *μ* as a function of *N*_s_, Matthiessen’s rule is applied to combine a constant mobility contribution *μ*_const_ and an *N*_s_-dependent contribution *μ*_n _~ 1/*N*_s_ (Fig. 3a)^25, 26^. This model approach has been used, in part, by Tanabe *et al*.^27^ to describe electrical Hall effect results for *μ* versus *N*_s_, where *N*_s_ is varied by electric field-effect doping. In Tanabe *et al*., *μ*_const_ represents a constant contribution from charged impurity (long range) scattering, and *μ*_n_ represents the *N*_s_-dependent point (short range) scattering contribution^27^. In contrast with our work, the ambient conditions were not varied in the experiments performed by Tanabe *et al*. Therefore, our experiment may include different contributions to charge carrier scattering and further experimentation is needed to determine the exact source of mobility limitation. Furthermore, the fabricated Hall bars and electrodes in Tanabe *et al*. may introduce additional contributions to carrier scattering compared to our virgin sample. In Tanabe *et al*., the number of charged impurities is likely not to vary throughout the experiment, unlike this work in which impurities can be introduced by gases. Our observation suggests that it is possible only the carrier density in the graphene changes as a result of gas exposure and where the observed change in mobility is only given by the *μ*(*N*_s_) dependence. This would also provide a possible explanation why both *μ*(*N*_s_) and σ(*N*_s_) (Fig. 3(a) and (b)) show so little dependence on the gas species. A variation in number of charged impurities during our experiment could also explain the change in *μ*, if number of charged impurities is proportional to *N*_s_^24, 28–33^. Other possibilities for the observed *μ* change include acoustic phonon scattering^34^, or even gas-induced modifications to interactions between graphene, buffer layer, or SiC substrate. The best-match model parameters for *μ*(*N*_s_) are *μ*_const_ = (3214 ± 35) cm^2^/Vs and *a* = (1.20 ± 0.03) × 10^16^ (Vs)^−1^, where *a* is the proportionality constant in *μ*_n _= *a*/*N*_s_ clearly describe all observed data points. Note that parameter *a* does not necessarily correspond to any specific scattering mechanism, but is reported here to describe the observed relationship.

Recent electrical Hall bar experiments on very similar epitaxial graphene also show a linear *μ* versus *N*_s_ dependence^35^. In the report by Yager *et al*., many Hall bar devices were fabricated and investigated on primarily 1 ML graphene. In contrast to our work, the variation in *N*_s_ reported by Yager *et al*. is a result of variation of small 2 ML coverage inside certain devices. In Ref. 35 it is noted that one would never know why the results are so inconsistent, unless microscopic images of the devices are inspected and variations in 2 ML coverage are detected. One advantage of the THz-OHE, and ellipsometry in general, is the ability to acquire an average response over the entire sample. This eliminates parameter variations associated with fabricating devices to obtain consistent values of *N*_s_, *μ*, and carrier type.

## Conclusions

In summary, we have obtained *N*_s_ and *μ* of epitaxial graphene as a function of gas exposure using the cavity-enhanced *in-situ* THz-OHE, which allows contactless determination of these properties. In addition to the free charge carrier properties, the THz-OHE allows access to the majority carrier type, which is determined to be electrons. The results of the best-match model analysis reveal important information about the ambient doping and scattering mechanisms for epitaxial graphene on Si-face (0001) 4H-SiC. In agreement with previous works^5, 6^, it is found that exposure to an inert gas, such as helium or nitrogen, reverses the electron withdrawal caused by ambient. The change in *N*_s_ and *μ* as a function of gas exposure can be approximated by a single process exponential decay function. The results indicate epitaxial graphene on Si-face SiC could take longer than one day to reach its final ambient state. This is important to consider when studying graphene recently exposed to ambient, since its properties may differ from day to day.

The observed *μ* versus *N*_s_ dependence is universal for all phases of gas exposure. This suggests inert gases reverse the scattering mechanism influence caused by air exposure. When a model is applied for *μ* as function of *N*_s_, we find one constant and one ~1/*N*_s_ term is needed to describe the dependence. This suggests that it is possible only the carrier density in the graphene changes as a result of gas exposure and where the observed change in mobility is only given by the *μ*(*N*_s_) dependence. This scenario also provides explanation for the observed universality of *μ*(*N*_s_) and σ(*N*_s_) dependencies on the gas species. However, further investigation is needed to distinguish the exact mechanisms which control and limit *μ*. The experiments performed here demonstrate the ability of the *in-situ* THz-OHE to obtain *N*_s_, *μ*, and majority carrier type with a high time resolution which is valuable for characterizing two-dimensional materials. Examining the relationship between these properties enables insight into the doping processes and scattering mechanisms as function of environmental variables.

## Methods

### Cavity-enhanced terahertz optical Hall effect

The measurement technique implemented here is the cavity-enhanced terahertz optical Hall effect. Recently, this technique has been demonstrated as viable non-contact method to obtain free charge carrier properties using low-field permanent magnets^18, 19^. A tunable, externally-coupled cavity is used to enhance the THz-OHE signal, which allows the accurate determination of a sample’s free charge carrier properties even at low magnetic fields. Placing the sample near the THz reflective metal surface of the permanent magnet allows the typically unused radiation emitted out the backside of the sample to be reflected back in (Fig. 1b), thus enhancing the THz-OHE signal. Model simulations are used to determine the ideal external cavity size, which for this graphene sample is 100 *μ*m.

### Gas flow cell

The body of the gas flow cell (Fig. 1a) is constructed from Delrin, and the top and bottom lids are acrylic. The gas cell window material is homopolymer polypropylene and each window is 0.27 mm thick. The flow rate used in the cell is approximately 0.5 liters/minute. The background pressure for all gas exposure phases was 1 atm. A vacuum pump (Linicon) is used to flow unaltered ambient gas into the cell, and pressurized purge lines are used to provide nitrogen and helium flow. The permanent magnet mounted inside the cell is high-grade neodymium (N42). The magnetic field near the north surface is 0.55 Tesla, which is determined using a Hall effect sensor (Lake Shore). THz radiation shielding (not shown) is used to suppress edge reflections from side walls, sample boundaries and magnet surfaces. The THz beam spot covers the entire sample. Any radiation incident on the edges of sample is suppressed by the THz radiation shielding.

### Terahertz ellipsometer

Measurements were performed using a custom-built rotating-analyzer THz ellipsometer at the Terahertz Materials Analysis Center in Linköping University^12, 19^. The instrument is capable of measuring the upper-left 3 × 3 block of the Mueller matrix, which fully characterizes the THz-OHE signal.

### Sample growth and characterization

Monolayer graphene is grown by high-temperature sublimation in Ar atmosphere on Si-face 4H-SiC^12, 36^. The representative 532-nm-wavelength reflectance-converted-thickness map (Fig. 1c) is obtained with a 300 nm lateral step resolution and a 100× objective^37^. The sample dimensions are 10 × 10 × 0.35 mm.

### Ellipsometry and optical Hall effect model analysis

In this work, ellipsometry is used to determine graphene’s free charge carrier properties. This is a technique, which measures polarization changes in electromagnetic radiation upon, in this case, reflection off a sample^38^. Ellipsometry is an indirect measurement technique that requires an optical model be fit to experimental data to obtain desired sample parameters. The fit algorithm used is the Levenberg-Marquardt non-linear regression method^39^. Here, ellipsometric data are reported in the Mueller matrix formalism^40^.

The sample’s optical response is governed by the dielectric function. In the THz spectral range, the dielectric function consists of contributions from free charge carriers and a magnetooptic contribution, which is due to the presence of magnetic field, as described in refs 41 and 42.

A stratified layer model is used to represent the graphene/SiC substrate/external cavity/metallic magnet surface system (Fig. 1b). The millimeter wavelength of the THz beam renders it insensitive to non-electrically conductive thin films of few nm thickness only. Hence, any intricate dielectric variations near the graphene-SiC interface, such as the formation of a buffer layer, is undetectable by our present ellipsometry setup. On the contrary, free charge carriers produce very large dielectric responses, and are clearly detectable. Here, a 1 nm thick layer containing free charge carriers is used to represent the graphene atop the SiC substrate with no intermediate layers. The SiC substrate is insulating and no free charge carrier contribution is included for this layer. In the same vain, a water film is not included in the THz optical model either since previous experiments for similar samples and for similar environmental conditions have shown water adsorbates on epitaxial graphene on SiC to be approximately 1 nm thick^15^. Using previously determined dielectric constants for water at THz frequencies^43^, our simulations show the change in polarization due to such a film can be neglected. This also implies that the inclusion of a water and/or buffer layer would have no effect on the accuracy of the reported *N*_s_ and *μ* values. Furthermore, the response of the bare Si-face SiC substrate as well as a buffer layer sample mounted on the permanent magnet were investigated by exposing to N_2_ and unaltered ambient gas. No change in the measured data is observed when switching between the gases.

To obtain the parameters *N*_s_ and *μ*, the optical model was fit to the *in-situ* data acquired at a single frequency (*ν* = 428 GHz). The parameters were determined by a point-by-point analysis, meaning *N*_s_ and *μ* were determined by a fit process to the experimental data obtained for each point in time separately.

Due to experimental constraints, effective mass *m* ^*^ is not obtained in the analysis and has been implemented as function of *N*_s_ using the equation ${m}^{\ast }=\sqrt{({h}^{2}{N}_{{\rm{s}}}\mathrm{)/(4}\pi {v}_{{\rm{f}}}^{2})}$, where *ν*_f_ = 1.02 × 10^6^ m/s is the Fermi velocity, according to Ref. 44. The cavity-enhanced THz-OHE is, in fact, capable of attaining m* in addition to *N*_s_ and *μ* ^18^. However to improve the time resolution of the *in-situ* measurement, experimental data was only obtained at one frequency *ν* = 428 GHz, one angle of incidence Φ_*a*_ = 45°, and one sample-magnet air gap distance *d*_gap_ = 100 *μ*m. If one or more of these variables were utilized during the *in-situ* measurement the sensitivity to all free charge carrier properties would increase.

During data analysis, to correct for slight misalignment of the THz ellipsometer we employ one frequency- and time-independent correction matrix in the optical model. The correction is made by matrix multiplication of the Mueller matrix representing the sample-magnet system and a correction matrix representing a small constant offset induced by the instrument. To find the correction matrix values used in the analysis, all the elements of that matrix were fit to data obtained at multiple frequencies (385 to 395 GHz) before the gas flow experiment. In this spectral range, there are no changes due to the sample or magnet, but only changes from minor instrument non-idealities.

### Experimental data

**Figure 4:**
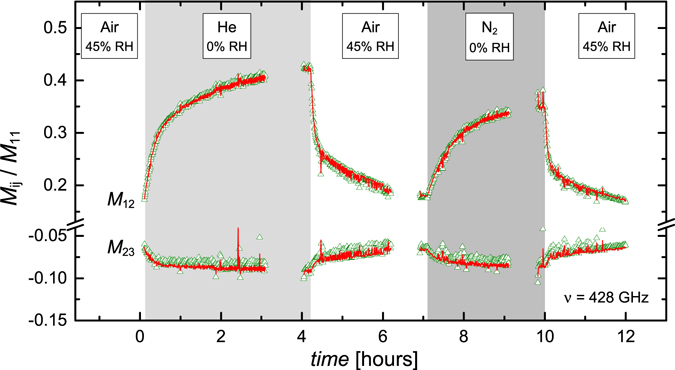
*In-situ* experimental (green triangles) and modeled (red lines) Mueller matrix data as a function of time for all gas exposure phases. The shaded regions correspond to different types of gas flow at various relative humidities (RH). Data shown here is acquired at a single frequency *ν* = 428 GHz. Mueller matrix elements not shown here are either similar or identical to the elements depicted and are excluded for brevity. Data is acquired at an angle of incidence Φ_*a*_ = 45° and at room temperature.

Figure 4 shows changes in the Mueller matrix data acquired at a single frequency (*ν* = 428 GHz) as a function of time for different gas exposures. The Mueller matrix elements depicted here are *M*_12_ and *M*_23_. For this sample and permanent magnet configuration, the *M*_12_ element experiences the largest change with variation in the free charge carrier properties. The frequency *ν* = 428 GHz was chosen because this is where the maximum change in *M*_12_ occurs. The *M*_23_, as well as all other off-block-diagonal Mueller matrix elements, characterize the THz-OHE signal and would be zero if there were no magnetic field present. The negative value of this element indicates the sample is n-type, since the sample is mounted on the north pole-face of the permanent magnet. Note that a sign reversal of this element would indicate a reversal of conductivity from n-type to p-type. Such type of change, for example, can be induced by use of reactive gases such as NO_2_^16^.

Breaks in the experimental data near the end of each gas flow phase are where data was obtained at multiple frequencies to verify the optical model. This is verification was done before the point-by-point analysis was performed. Data recorded prior to those reported here were taken during repeated gas exposure cycles, and were found highly reproducible. Note, certain Mueller matrix elements are excluded for brevity. The *M*_21_ and *M*_32_ are omitted due to being essentially equal to *M*_12_ and *M*_23_ respectively. *M*_13_ and *M*_31_ are essentially equal and are omitted since they resemble *M*_23_. *M*_22_ and *M*_33_ show very little change and are omitted.
